# Feasibility of a Systematic, Comprehensive, One-to-One Training (SCOOT) program for new scooter users: study protocol for a randomized control trial

**DOI:** 10.1186/s13063-017-1963-y

**Published:** 2017-05-25

**Authors:** W. Ben Mortenson, Sharon Jang, Charlie H. Goldsmith, Laura Hurd Clarke, Sandra Hobson, Richelle Emery

**Affiliations:** 1 The Department of Occupational Science and Occupational Therapy, University of British Columbia, T-325-2211 Wesbrook Mall, Vancouver, BC V6T 2B5 Canada; 2 GF Strong Rehabilitation Center, 4255 Laurel Street, Vancouver, BC V5Z 2G9 Canada; 3 Mary Pack Arthritis Center, 895 West 10th Ave., Vancouver, BC V5Z 1L7 Canada; 4 University of British Columbia, 1924 West Mall, Vancouver, BC V6T 1Z2 Canada; 5 School of Kinesiology, University of British Columbia, 1156-1924 West Mall, Vancouver, BC V6T 1Z2 Canada; 6 University of Western Ontario, 1201 Western Road, London, ONT N6G 1H1 Canada; 7 Vancouver Coastal Health – Community Care, 520 West 6th Avenue, Vancouver, BC V5Z 1A1 Canada

**Keywords:** Scooter, Training, Randomized control trial, Learning

## Abstract

**Background:**

Mobility scooters can facilitate community participation among individuals with mobility limitations. However, accidents are a serious concern with scooter use. Scooter training has been recommended to improve safety, but there are currently few validated programs available. Therefore, we developed a Systematic, Comprehensive, One-to-One Training (SCOOT) program for scooter users. We will conduct a study to evaluate the outcomes produced by the provision SCOOT.

**Methods:**

This feasibility study will use a mixed-methods, rater-blinded, randomized control trial, with a two-step wedge design. The study has two arms: an immediate intervention group, which will receive the intervention directly after baseline assessments, and a delayed intervention group, which will receive the intervention after a 6-week period. Forty participants, who will be stratified based on whether or not participants have previously held a driver’s license, will be randomly assigned to each arm. The intervention for this study consists of 6 weeks of one-to-one scooter training by an experienced occupational therapist, who will provide training once or twice per week over the 6 weeks. The primary outcome measure is subjective scooter skills, measured using the Wheelchair Skills Test for scooters. Secondary outcomes include objective scooter skills, confidence, mobility, and satisfaction with selected participation activities. Descriptive measures include cognitive status, functional status, hearing, vision, physical accessibility of the home and community, and visual attention and task switching. Qualitative interviews will be conducted with the first ten willing participants from each group to learn about their scooter use and experiences with SCOOT.

**Discussion:**

The results of this study will inform a larger randomized control trial. If the intervention is proven to be effective in this larger study, it may have important implications for policy and practice.

**Trial registration:**

ClinicalTrials.gov identifier: NCT02696213. Registered on 23 February 2016.

**Electronic supplementary material:**

The online version of this article (doi:10.1186/s13063-017-1963-y) contains supplementary material, which is available to authorized users.

## Background

Many people have disabilities that necessitate the use of powered mobility devices. Mobility scooters (i.e., three or four wheeled devices controlled by a tiller) may be preferred over power wheelchairs, as they are generally more affordable and perceived as less stigmatizing [1, 2]. Although the prevalence of scooter use has been increasing in North America [3, 4], scooter use appears to vary considerably in different countries. For example, it was estimated that 0.3% of Canadians used a scooter [4], whereas in Australia this figure was 1% [5].

Scooters, like many mobility devices, may be a mixed blessing [1]. Several studies have found that the provision of a mobility scooter is positively associated with feelings of independence, higher frequency of daily activities, and increased social participation [6–9]. However, scooter-related accidents (e.g., falls, collisions with stationary and moving objects) are a concern, as they can cause serious, sometimes fatal injuries to users and others [10, 11]. A wide spectrum of injury rates have been reported for scooter users (e.g., from 1.54 [12] to 15 [5] injuries per person per year). Similarly, a variety of accident rates have been reported for power wheelchair users (e.g., 5–18% of community dwelling users experience accidents each year [13]). To improve safety, scooter training has been recommended [14], which could include advanced skills such as navigating curbs and uneven surfaces, accessing public transit and elevators, and avoiding obstacles [15].

There are a variety of potential benefits of wheeled mobility skills training but few users report receiving formal training. Manual wheelchair training has been found to improve skills [13] and increase confidence [16]. However, most scooter users receive very little training, which may be attributed to limited accessibility. Two surveys found that a quarter of scooter users received training [5, 15]. Although there appears to be great variability in terms of the training provided [15], training appears to focus on rudimentary skills (e.g., basic operating skills, driving indoors and outdoors, transferring on and off the scooter), rather than more advanced skills such as crossing streets or using transportation [15].

Research on the efficacy of scooter training is limited. In a scoping review conducted by Mortenson and Kim [17], two small scale, randomized control trials (RCTs) were identified. One trial found that 3D virtual reality training in combination with conventional training produced similar improvements in scooter skills as conventional training alone. The second study found that scooter skills improved significantly with meta-cognitive training combined with on-road driving practice, compared to computerized cognitive training alone [18]. However, the validity of these findings are threatened by their small sample sizes, lack of non-intervention control arms, and use of non-validated outcome measures [17], such as the study-specific functional evaluation rating scale used in the study by Jannink et al. [19].

To improve the mobility and social participation of scooter users and to decrease safety concerns, we created a community-based training program built on the Wheelchair Skills Training Program [20] and feedback from Vancouver Coastal Health scooter prescribers, called Systematic, Comprehensive, One-to-One, Training (SCOOT) for scooters. We are conducting a study to explore the feasibility of a mixed-methods RCT, which will evaluate the efficacy of this intervention. Based on the typology of feasibility research [21], this study will focus on process assessment (i.e., recruitment rate, retention rate, treatment fidelity, adherence rate, suitability of the eligibility criteria and measures, and respondent burden) and on scientific assessment (i.e., safety of the intervention, reliability and validity of the measures with this population, estimates of the effect of the intervention and variances of the effects).

### Hypothesis

We anticipate that the feasibility outcomes will support a subsequent multi-site trial. We expect that our recruitment targets will be met, that > 90% treatment, > 80% adherence, and > 80% retentions will be obtained, that SCOOT will be as safe as non-intervention, and that participants will provide complete responses to > 90% of items from all measures.

### Objectives

#### Quantitative objective

To evaluate the outcomes produced by the provision of a comprehensive program of scooter training among older adults.

#### Qualitative objectives

To explore how this intervention is experienced by recipients and trainers and to understand how the intervention was implemented in context.

## Methods

### Study design

**Fig. 1:**
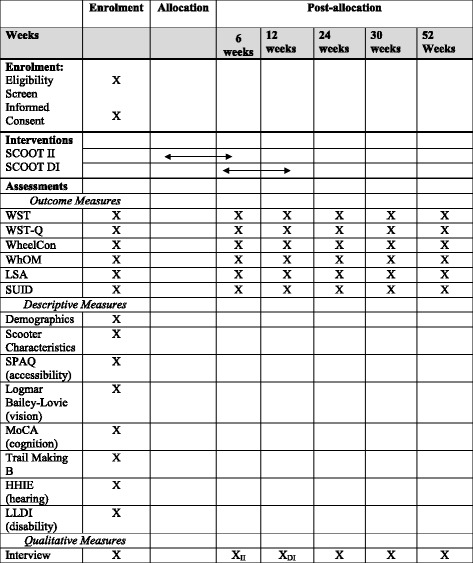
SPIRIT figure. *II* Immediate Intervention, *DI* Delayed Intervention, *WST* Wheelchair Skills Test for Scooters, *WST-Q* Wheelchair Skills Test – Questionnaire for Scooters, *WheelCon* Wheelchair Use Confidence Scale, *WhOM* Wheelchair Outcome Measure, *LSA* Life Space Assessment, *SUID* Scooter Use Incidents Diary, *SPAQ* Scooter Physical Accessibility Questionnaire, *MoCA* Montreal Cognitive Assessment, *HHIE* Hearing Handicap Inventory for the Elderly, *LLDI* Late Life Disability Index

To conduct this exploratory, mixed-method, rater-blinded RCT, a two-step wedge design will be used, as illustrated in Fig. 1. With a step-wedge design, participants are randomly assigned to receive the same intervention at different times [22]. For this study, a total of 40 participants, stratified based on whether or not they have previously held an automobile driver’s license, will be randomly assigned to either the immediate intervention group or the delayed intervention group. As illustrated in Fig. 1, the immediate intervention group will receive the intervention after consent and baseline data have been collected, while the delayed group will receive the intervention after a 6-week delay. The primary endpoint of this study is at 6 weeks, so we can compare the effects of the intervention with the group who has yet to receive it.

### Randomization

The allocation of each participant will be communicated to the study research coordinator after baseline data and consent have been obtained. The allocations will be managed by the study statistician using an auditable computer program and a 1:1 allocation ratio within each stratum. Blocking sizes will not be revealed until needed for analysis.

### Ethics

Ethical approval for this study has been provided by the University of British Columbia (H15-09121) and by the Vancouver Coastal Health Authority (V15-09121). This study was funded through a grant from the Canadian Institute of Health Research (340545). This protocol paper has been written in accordance with the SPIRIT (Standard Protocol Items: Recommendations for Interventional Trials) guidelines (Additional file 1) [23]. Any modification of important protocol will be made through an ethics amendment with the institutional review board prior to implementing the given change.

### Participants

For this feasibility study we plan to recruit 40 participants. As the majority of wheeled mobility users are older adults [4], the eligible community dwelling participants for this study are (1) English-speaking adults, (2) aged ≥ 60 years, (3) have one month or less combined experience of using a scooter within the past year, and (4) are able to independently transfer in and out of a scooter. We have not defined the population of interest based on their diagnoses (e.g., stroke, spinal cord injury, Parkinson’s disease), because the use of a scooter is not diagnosis specific. Individuals who have cognitive impairments that will prevent them from providing consent and from reliably completing the study questionnaires, reside in a nursing home, or plan to move outside the study intervention delivery area within the next year will be excluded.

Participants will be recruited through a variety of sources, including community healthcare units, vendors, and various seniors’ groups and seniors’ centres. Participants will be recruited through these organizations via posters, presentations, social media, and referrals. Occupational therapy staff and vendors will invite eligible clients to consider the trial.

### Procedure

The research coordinator and principal investigator will train raters to administer all study measures. This training will include review of manuals, observations of pre-recorded mock interviews and assessments with practice scoring, practice administration with other research staff with feedback provided, and video recording of practice administration for self-reflection.

Upon enrolment, participants will be provided with a unique ID number, and the researcher will obtain written consent prior to baseline testing with all participants. The immediate intervention group will receive SCOOT after their baseline testing once or twice per week over a 6-week period (varying from 9 to 18 hours total), while the delayed intervention group will receive SCOOT 6 weeks after baseline testing. Upon completion of the intervention, all participants will be re-assessed at 6, 12, 26, and 52 weeks after randomization. Participants will also be asked not to participate in any additional training until 12 weeks have elapsed. The intervention will be discontinued upon the participant’s request, or if they are unable to continue with the research due to unforeseen events (e.g., illness).

Measures will be administered in a random order to reduce order effects. All measures, except the Wheelchair Outcome Measure (WhOM), will be collected by a blinded research assistant. The WhOM will be administered by the study therapist as this will inform the intervention. During training sessions, the therapist delivering the intervention will record any adverse effects of the intervention such as falls. As suggested by Little et al. [24], to reduce missing data, we will stress, during the consent process and whenever there is contact between participants and study personnel, the importance of collecting measurement data for the duration of the study, especially for those who discontinue the intervention.

### Experimental intervention

SCOOT is a community-based, client-centered intervention that embeds skills training into social activities that users want to accomplish. It also uses trainer-facilitated problem solving to identify strategies to manage environmental barriers and to enable better user social participation. This intervention represents a departure from customary scooter training that is often very limited and which focuses on learning discrete skills outside of the user’s normal environment. With SCOOT, skill training (i.e., on driving, basic and advanced scooter skills) will take place during self-selected, home- or community-based activities that participants want to perform using their scooters. We will identify these activities using the WhOM [21], which identifies the most important home- and community-based activities participants wish to perform using their scooters (described below). The skills training component of the intervention is based on the scooter version of the Wheelchair Skills Training program [20].

The intervention will be held in the neighborhood of the participants, located in the Greater Vancouver Regions District of British Columbia, Canada. The intention of conducting the study within the neighborhood of the participant is to facilitate the transferability of training and to improve the ecological validity. In addition, training in the community aims to reduce participant burden of travelling to our research site, and to improve adherence. These neighborhoods are all located within an urban setting, and locations include, but are not limited to, community centers, grocery stores, and parks.

SCOOT for scooter mobility skills will be provided by occupational therapist trainers who will be trained by the principal investigator to use the same standardized approach. The therapist will keep a record of the skills that they have taught and hours of training provided. During training sessions, the therapist delivering the intervention will record any adverse effects of the intervention such as falls. We will collect data on the number of therapist visits, length of visits, therapist years of practice, years of experience working in this area, and therapist qualifications.

### Outcome measures

#### Primary outcome measure (subjective skill capacity)

The subjective version of Kirby et al.’s Wheelchair Skills Test [20] for scooters incorporates three domains of skills training, namely skill capacity, confidence, and performance. For this study, the primary outcome measure will be perceived capacity. It consists of 29 items that are scored by the participant from 0 to 2, where 0 = unsafe or unable, 1 = safe with difficulty, and 2 = safe without difficulty; a percent score is calculated by dividing the total score by the number of applicable items and multiplying that number by 100. The measure assesses basic indoor mobility skills (driving forward and backwards), transfers on and off the scooter, and outdoor driving skills, including maneuvering, curbs, and ramps. The intraclass correlation coefficient (ICC) for 2-week test-retest reliability of the Wheelchair Skills Test among power wheelchair users is 0.78 (95% confidence interval, 0.68–0.86) [25]. As scooters and power wheelchairs are both motorized mobility devices, a similar reliability among scooter users is expected.

#### Secondary outcomes (body structure and function and activity and participation levels)

##### Objective scooter skills

Objective scooter skills will be measured using Kirby et al.’s Wheelchair Skills Test [20]. The test consists of 29 rater administered items that include operating the scooter and indoor and outdoor scooter skills, similar to the subjective version. The ICC for the test-retest reliability of the objective version of the measure among 20 experienced scooter users is 0.90 (95% confidence interval, 0.74–0.95) [26].

##### Satisfaction with participation in selected activities

The WhOM is a participant-specific tool that evaluates satisfaction with participant-identified home and community activities associated with wheeled-mobility provision using an 11-point scale (0 = completely unsatisfied to 10 = completely satisfied) [21]. A mean satisfaction score is calculated by dividing the sum of all satisfaction scores by the number of goals. The WhOM demonstrates promising psychometric properties among community-dwelling, power-mobility users with good 2-week test-retest reliability (ICC = 0.91 for the mean satisfaction score) [27] and moderate correlations with other measures of social participation and device satisfaction [28].

##### Mobility

The Life Space Assessment [29] is a self-report measure of participants’ frequency and independence of mobility in increasingly larger life spaces (e.g., within their (1) home, (2) yard, (3) neighborhood, (4) city or town, and (5) beyond) over the past month. Frequency is measured on a four-point scale: 1 = less than once per week; 2 = 1–3 times per week; 3 = 4–6 times per week; and 4 = daily. Independence is measured on a three-point scale (1 = personal assistance required; 1.5 = assistive device used; 2 = independent). A total composite life-space score, which varies from 0 to 120, can be calculated by multiplying the frequency by the independence by the weighting for each life space (1–5) and adding these together. Among power-mobility users (scooter and power-wheelchair users), the 2-week test-retest reliability is high for the composite score (ICC = 0.87) [30]. Life-space mobility has been found to be significantly higher for those who have had their power-mobility devices for six months or longer [31].

##### Scooter skills confidence in the social environment

We will use the shortened version of the social environment scale of the Wheelchair Use Confidence Scale for manual wheelchair users [32] that was developed by Sakakibara [33] to assess scooter users’ confidence negotiating their social milieu. For this study the word wheelchair was replaced with scooter. The scale uses an 11-point scale (0 = not confident to 10 = completely confident) and a total mean score between 0 and 100 can be calculated by finding the sum of the scores of each item.

##### Scooter use and incidents

Participants will be asked to keep a diary of any scooter-related adverse events, including the following: tips or falls from the scooter, injuries to self, accidental contact with others, injuries to others, and damage to property. Participants will be asked to record the number of hours per day that they use their scooters over the course of the study.

#### Descriptive measures and covariates (body function, health, environmental, and personal factors)

Descriptive data will be collected that includes participants’ socio-demographic characteristics (age, sex, income level of education, ethnic origin, language, marital status, type of dwelling, diagnoses, duration of functional problems, previous experience driving vehicle(s), and amount of formal care-giving received, if any). Although randomization should control for differences between the treatment groups, we will collect data on the following constructs that could affect scooter-user outcomes and which will provide additional descriptive information. The data then may be used as covariates, if there are important differences between the groups at baseline.

##### Cognitive status

The Montreal Cognitive Assessment [34], a widely used, cognitive test with high test-retest reliability (*r* = 0.92), has a better ability to detect mild cognitive impairment than the Mini-mental Status Exam [35]. Scores on the test vary from 0–30, where scores of 26 or above are considered normal.

##### Functional status

The activity-limitation domain of the Late Life Functioning and Disability Measure-Computer Assisted Testing (Version 1.04) (LLFDI-CAT) [36] will be used to measure the participant’s self-perceived, physical function. Drawing from a bank of 141 items, CAT continues until a standard error of 3 or less is obtained for the domain score, or a maximum of 10 items has been administered. The activity-limitation domain demonstrated high test-retest reliability (*r* = 0.85) among a sample of 102 community-dwelling older adults; it was moderately correlated (*r* = 0.72) with the physical component summary score of the Veteran’s Rand-36 [37]. The activity-limitation domain has two sub-scales: (1) basic mobility and handling and (2) daily activities. Standardized scores vary from 0 to 100, where the mean score is 50 and lower scores are indicative of greater limitation.

##### Hearing

We will use the Hearing Handicap Inventory for the Elderly Screening Version to measure hearing disability [38]. It is a 10-item self-report questionnaire. Scores vary from 0 to 40, and scores above 26 suggest an important hearing handicap. Its psychometric properties have been demonstrated across multiple studies [39].

##### Scooter characteristics

We will collect detailed information about the participant’s scooters including the number of wheels, smallest turning radius, size of wheels, length, width, and clearance.

##### Scooter physical accessibility of participant home and community

This will be measured by using 14 dichotomous (yes/no) questions, where no is scored as a 0 and yes is scored as a 1. Questions were derived in part from those asked in a study about environmental accessibility factors related to wheelchair use [40] and the Usability Scale for Assistive Technology-Wheeled Mobility version [41]. Higher scores indicate greater accessibility, which may facilitate increased mobility and social participation.

##### Visual attention and task switching

Trail Making B [42] will be used to measure visual attention and task switching. For this measure, the time required to draw lines between a sequential pattern of numbers and letters is recorded. The measure demonstrates high test-retest reliability (r = 0.95) [43]. Scores on the measure are predictive of on-road automobile driving performance [43].

##### Visual acuity

The Snellen eye chart [44] will be used to measure visual acuity. We will convert Snellen scores to the Logarithm of the Minimum Angle of Resolution (LogMAR) by taking the negative log of the Snellen score in decimal form [45], as LogMAR is recommended for statistical analyses [46]. LogMAR is based on a logarithmic scale (from 0 to 1), where 1 is indicative of perfect vision.

### Qualitative methods

Interviews will be conducted (1) at baseline prior to scooter training, (2) at 6 weeks, (3) at 6 months, and (4) at the end of the study. The initial interviews will focus on how participants currently use their scooters and the concerns they have, the second interviews will focus on how the intervention was experienced, and the final interviews will focus on longer-term outcomes of scooter use. We will interview the first ten interested participants in each group. Additional participants will be selected purposefully, depending on the analysis of the data from these first participants. These participants will be selected to elucidate themes emerging from the data from the first participants, and to understand negative cases better. For example, if one participant reacts negatively to the intervention, we will interview additional participants with similar demographic profiles.

### Analysis

#### Quantitative data

Data will be input into a password protected document and will be double checked for accuracy. Descriptive statistics (e.g., means, frequencies, proportions, standard deviations) and distributions and box plots will be used to display all study data. The percentage of skills taught will be calculated to determine the treatment fidelity, and the percentage of sessions attended will be calculated to determine adherence. These quantitative data will be supplemented with data from the qualitative portion of the study. We will explore the use of multiple imputation for missing outcome measure data [47]. A detailed statistical analysis plan to handle various statistical analysis issues will be created [48]. To identify covariates that will be controlled for in the larger study, we will identify factors that are strongly related to the primary and secondary outcome measures. To determine the effect size for the primary and non-count secondary outcome measures, we will perform various models, controlling for baseline scores, using an intention-to-treat analysis. That is, all participants will be included in the group to which they were allocated for purposes of analysis, whether or not they completed the intervention for that group. As this is a feasibility study, we will also calculate effect sizes based on the intervention received (e.g., on an as-treated basis). Using G*Power 3.1.0, this sample size should give us the ability to detect a large effect size of 0.46 (with α = 0.05 and power = 80%). For count data (e.g., use, accidents, and falls), we will determine the effect size by using Poisson regression [49].

#### Qualitative data

Audio files will be transcribed verbatim and will be anonymized by replacing any proper nouns with pseudonyms to protect the identity of the participants. Based on the process outlined by Thorne et al. [50, 51], we will read and re-read the data to identify key concepts based on recurring, converging, and contradictory patterns. In addition, themes and illustrative examples will be identified during this process. We will develop broad categories to organize and inductively code the raw data. Codes within and across participants will be developed through this iterative. Example codes will be compared between interview transcripts. Any “negative cases” that do not fit with conceptual understandings of the data will be explored to develop explanations for the observed variability. Ultimately, codes will be grouped into relevant themes and organized in a manner that is intended to promote understanding of how the SCOOT intervention was experienced, to contextualize understandings about how the intervention is implemented, and to determine how SCOOT affected participants.

## Discussion

Although mobility training is generally thought to enhance users’ skills, daily activities, and social participation, there is little research evidence to support these assumptions [20]. We expect that the feasibility outcomes will be strong enough to support the conducting of a subsequent multi-site trial with a sufficient sample size to enable us to quantify definitive outcomes such as adverse events (e.g., injuries and abandonment). Furthermore, this feasibility study will inform research that can produce credible new knowledge describing multiple outcomes that users experience following SCOOT. It will also lay the groundwork for additional studies that examine the cost-effectiveness of this intervention and attempt to identify more economical ways of delivering this training, such as by peer mentoring, telehealth, or digital media.

If SCOOT is shown to be effective, it may have important practice and policy implications. It will enable service providers to offer evidenced-based scooter training for the first time. Policymakers can be approached to lobby for changes in the ways that scooter training is provided and funded. We will relay these findings to policymakers through our institutional collaborators. Additionally, we will submit manuscripts describing this work to high-impact, peer-reviewed journals and give presentations at international and national conferences. Furthermore, results will be shared with collaborating health organizations via in-services and workshops with staff, and by publishing a lay summary of the findings in organizational newsletters. Most importantly, by improving safety, decreasing injuries, preventing fatalities, and enabling social participation, it is anticipated that the SCOOT will have a powerful effect on the physical and mental health and quality of life of Canadians who rely on these devices and that of their families.

As a feasibility study for a RCT, the research has several limitations. First, given the single-site nature of this research, new feasibility issues may arise in attempting to apply the results to additional sites; however, findings from our feasibility study should be able to help us anticipate many of these problems so that they can be addressed proactively. Secondly, the size of the current sample only allows for the detection of large effect sizes. Larger samples may be required to detect smaller effect sizes, which may occur with important secondary outcomes such as accidents and injuries.

In summary, given the limited research on scooter training, this feasibility study is needed to lay the groundwork for a larger RCT to evaluate whether our novel, community-based intervention improves mobility, social participation, and safety. If the results of this larger experimental study are positive, we will perform additional research evaluating the best way to deliver this intervention, e.g., mentorship or online training. Economic studies can be performed to conduct a cost-benefit analysis of the intervention in the future.

## Trial status

This trial is currently actively recruiting for participants.

## Additional file

Additional file 1: SPIRIT 2013 Checklist: Recommended items to address in a clinical trial protocol and related documents^*^. (DOC 120 kb)

## Abbreviations

ICC: intraclass correlation coefficient
LogMAR: logarithm of the minimum angle of resolution
RCT: randomized control trial
SCOOT: Systematic, Comprehensive, One-to-One Training for scooters
WhOM: Wheelchair Outcome Measure
